# Metachronous Isolated Inguinal Lymph Node Metastasis at a Previous Hernia Repair Site following Potentially Curative Surgery for Transverse Colon Cancer: A Case Report

**DOI:** 10.70352/scrj.cr.25-0475

**Published:** 2025-12-24

**Authors:** Hiroyuki Fujimoto, Masatsune Shibutani, Yuki Seki, Hiroaki Kasashima, Tatsunari Fukuoka, Taiyo Otoshi, Junji Uchida, Kiyoshi Maeda

**Affiliations:** 1 Department of Gastroenterological Surgery, Osaka Metropolitan University, Graduate School of Medicine, Osaka, Osaka, Japan; 2 Department of Urology, Osaka Metropolitan University, Graduate School of Medicine, Osaka, Osaka, Japan

**Keywords:** colon cancer, inguinal lymph node metastasis, inguinal hernia, spermatic cord

## Abstract

**INTRODUCTION:**

Inguinal lymph node metastasis from colon cancer is extremely rare. Therapeutic strategies have not been established regarding preventive measures for hernia recurrence following mesh removal when the mesh used in inguinal hernia repair is infiltrated by a tumor. Similarly, no treatment strategies have been developed regarding the necessity of prophylactic orchiectomy when a tumor infiltrates the spermatic cord and requires resection.

**CASE PRESENTATION:**

We report a rare case of metachronous isolated inguinal lymph node metastasis following potentially curative surgery for transverse colon cancer. An 83-year-old male underwent laparoscopic colectomy with D3 lymphadenectomy for transverse colon cancer. At 1 year postoperatively, he presented with a painful mass in the left inguinal region. Imaging suggested transverse colon cancer recurrence in the inguinal lymph node. Diagnostic and therapeutic inguinal lymphadenectomy was performed. The tumor had infiltrated the previously placed mesh for a prior inguinal hernia repair and adhered to the spermatic cord. Therefore, en bloc resection of the mesh and spermatic cord was performed. No additional reinforcement was necessary because the structural strength of the inguinal region was preserved following mesh removal. Furthermore, orchiectomy was not performed owing to testicular atrophy due to advanced age. Histopathological examination confirmed inguinal lymph node metastasis of transverse colon cancer. At 4 months postoperatively, the patient remained free from colorectal cancer recurrence, testicular necrosis, or inguinal hernia recurrence.

**CONCLUSIONS:**

This case represents an extremely rare instance of metastasis occurring at the site of a prior inguinal hernia repair with mesh implantation. Considering the potential need for spermatic cord resection and mesh removal, appropriate surgical strategies for such scenarios should be included in preoperative planning.

## Abbreviations

CDX-2
: caudal-type homeobox 2
CEA
: carcinoembryonic antigen
CK-7
: cytokeratin 7
CK-20
: cytokeratin 20
FDG
: fluorodeoxyglucose

## INTRODUCTION

Inguinal lymph node metastasis from colorectal carcinomas is considered uncommon compared with more common metastatic sites, including the liver, lungs, peritoneum, and regional or para-aortic lymph nodes. Only a few cases have been reported in the literature,^1–10)^ and isolated inguinal lymph node metastasis is even rarer, with only a limited number of cases described.^1,9)^ Furthermore, therapeutic strategies have not been developed regarding preventive measures for hernia recurrence following mesh removal when a tumor infiltrates the mesh used in inguinal hernia repair. Similarly, no treatment strategies have been established regarding the necessity of prophylactic orchiectomy when the spermatic cord is infiltrated by a tumor requiring resection. Therefore, each case requires tailored management.

## CASE PRESENTATION

**Fig. 1:**
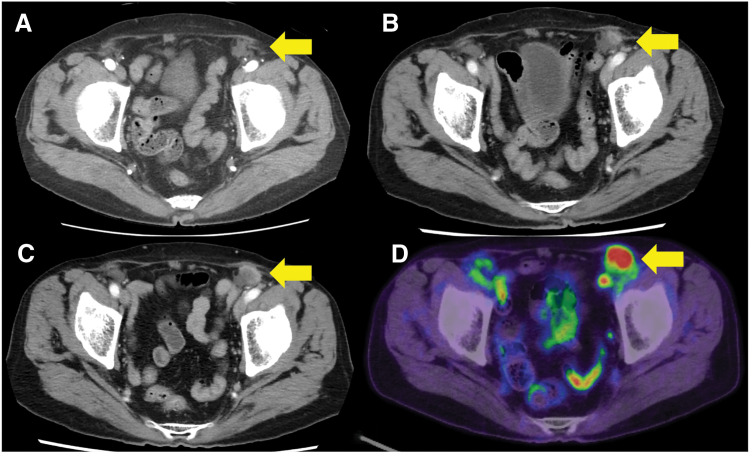
CT and PET–CT findings. (**A**) Preoperative contrast-enhanced CT for transverse colon cancer revealed only postoperative changes from prior inguinal hernia repair. The arrow highlights the absence of apparent metastatic nodules. (**B**) Contrast-enhanced CT at 6 months postoperatively demonstrated the appearance of an enhancing nodule in the left inguinal region. The arrow highlights the inguinal mass. (**C**) Contrast-enhanced CT at 1 year postoperatively showing interval enlargement of the left inguinal lymph node. The arrow highlights the inguinal mass. (**D**) PET-CT revealing fluorodeoxyglucose uptake in the left inguinal lymph node. The arrow highlights the inguinal mass.

An 83-year-old man underwent laparoscopic colectomy with D3 lymphadenectomy for transverse colon cancer (pT4a, N0, M0, Stage IIb). Adjuvant chemotherapy is known to be effective in patients aged ≥80 years.^11)^ Although this patient was old, his performance status was favorable; however, his pT4a status indicated a high risk of recurrence. Therefore, adjuvant chemotherapy was planned.^12)^ Additionally, fluoropyrimidine monotherapy has been reported to be safe in older patients, with a low incidence of severe adverse events.^13)^ Thus, adjuvant chemotherapy with tegafur–uracil/leucovorin was administered in the present case. However, following the development of Grade 2 liver dysfunction and Grade 2 gastrointestinal symptoms, both classified according to the Common Terminology Criteria for Adverse Events version 5.0,^14)^ the patient requested chemotherapy discontinuation, and the treatment was ceased following 3 cycles. His past medical history encompassed bilateral inguinal hernia repair via an anterior approach with mesh placement 13 years prior. Preoperative contrast-enhanced CT for transverse colon cancer revealed only postoperative changes from the previous inguinal hernia repair, with no findings suggestive of metastasis (**Fig. 1A**). However, a routine surveillance contrast-enhanced CT performed 6 months after colectomy demonstrated a newly developed 10 × 5 mm nodule in the left inguinal region (**Fig. 1B**). At the time of this examination, neither elevation of tumor markers nor any subjective symptoms were observed; therefore, a watch-and-wait approach was adopted. Furthermore, on the contrast-enhanced CT performed 1 year after surgery, the lesion had enlarged, appearing as a 20 × 16-mm enhancing mass in the left inguinal region (**Fig. 1C**). Physical examination revealed a tender, hard mass corresponding to the same location. PET-CT demonstrated intense FDG uptake at this site, with a maximum standardized uptake value of 14.6, while no abnormal uptake was detected elsewhere (**Fig. 1D**). Following potentially curative colectomy, CEA and cancer antigen 19-9 levels remained within the normal range during follow-up; however, upon inguinal mass detection, the CEA level increased from 3.7 to 7.9 ng/mL. Inguinal lymph node recurrence of transverse colon cancer was suspected on the basis of imaging findings and tumor marker trends. Inguinal lymphadenectomy was performed because the lesion was solitary.

**Fig. 2:**
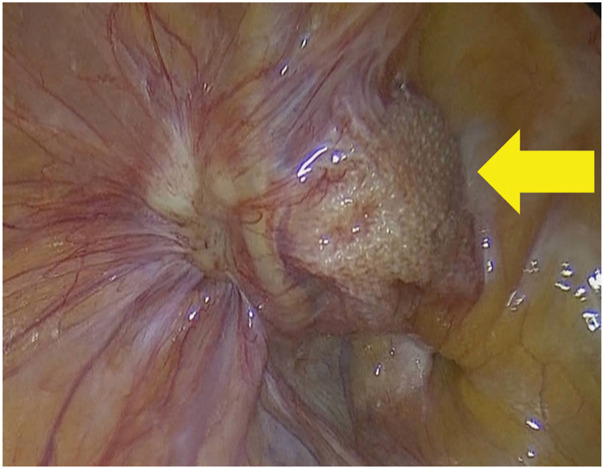
Laparoscopic findings. The arrow highlights the absence of a mass on the peritoneal side of the mesh and the absence of disseminated nodules in the surrounding area.

**Fig. 3:**
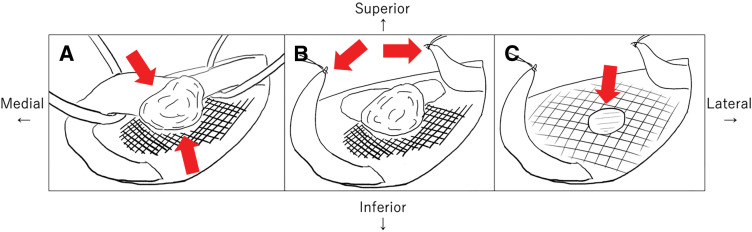
Intraoperative findings. (**A**) The arrows highlight the tumor strongly adherent to the spermatic cord and the onlay mesh placed during the previous hernia repair, making separation challenging. (**B**) Complete resection required en bloc removal of the mesh and the spermatic cord. The arrows highlight the transected end of the spermatic cord. (**C**) After mesh removal, the posterior wall of the inguinal canal maintained adequate structural strength due to surrounding tissue fibrosis; therefore, no additional reinforcement was necessary. The arrow highlights the mesh defect after removal.

Diagnostic laparoscopy was initially performed to rule out peritoneal seeding as a continuous disease process. No evidence of peritoneal dissemination or intra-abdominal organ metastasis was observed (**Fig. 2**). Subsequently, the mass was resected via an anterior approach. However, it was strongly adherent to the spermatic cord and the onlay mesh placed during the previous hernia repair, making separation challenging (**Fig. 3A**). Complete resection required en bloc removal of the mesh and spermatic cord (**Fig. 3B**). Following mesh removal, the posterior wall of the inguinal canal maintained adequate structural strength owing to surrounding tissue fibrosis; therefore, no additional reinforcement for hernia recurrence prevention was performed (**Fig. 3C**). Furthermore, following consultation with a urologist, the spermatic cord was resected; however, considering age-related testicular atrophy, the risk of testicular necrosis was considered low, and prophylactic orchiectomy was not performed.

**Fig. 4:**
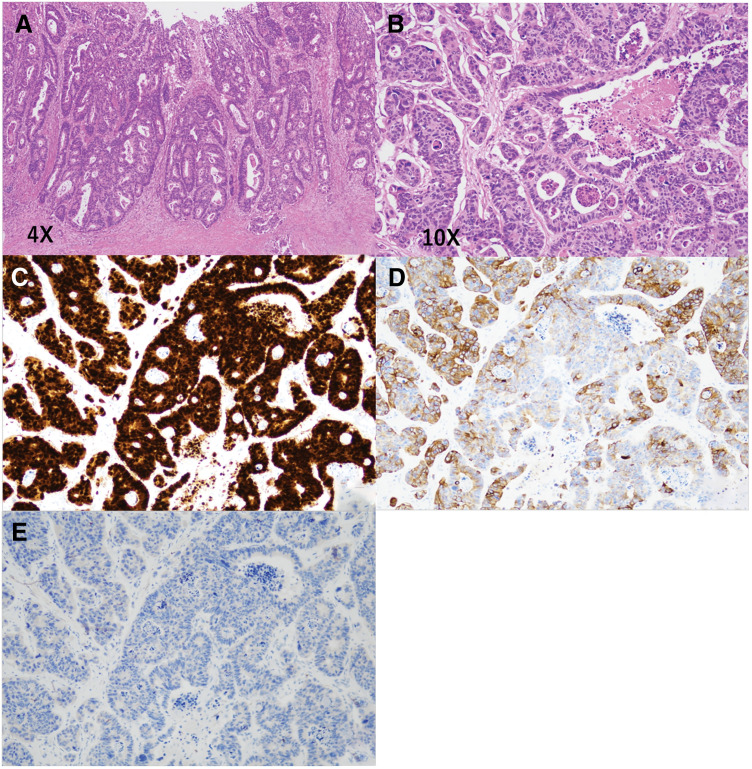
Histopathological and immunohistochemical findings. (**A**) Primary transverse colon adenocarcinoma. (**B**) Metastatic inguinal lymph node depicting preserved lymphoid architecture with infiltrative growth of adenocarcinoma forming glandular structures. (**C**) Positive staining result for caudal-type homeobox 2. (**D**) Positive staining result for cytokeratin 20. (**E**) Negative staining result for cytokeratin 7.

Histopathological examination revealed tumor histology comparable to that of the primary transverse colon cancer within the lymph node component. Immunohistochemical staining was positive for CDX-2 and CK-20 but negative for CK-7, supporting the diagnosis of inguinal lymph node metastasis from transverse colon cancer (**Fig. 4**). The tumor infiltrated the mesh; however, the vas deferens or the testicular vessels within the spermatic cord showed no invasion.

The postoperative course was uneventful; on POD 6, the patient was discharged. The patient did not want adjuvant chemotherapy owing to his experience with adverse events caused by adjuvant chemotherapy following potentially curative colectomy. Postoperatively, tumor marker levels promptly returned to normal. At the 4-month follow-up, no evidence of colorectal cancer recurrence, testicular necrosis, or inguinal hernia recurrence was observed.

## DISCUSSION

Inguinal lymph node metastasis is known to occur in pelvic malignancies and external genitalia cancers. However, reports of isolated inguinal lymph node metastasis are exceedingly rare. Besides gastrointestinal malignancies, only a few cases have been described in ovarian cancer, in which the metastatic pathway has been considered either lymphatic or hematogenous.^15,16)^ In gastrointestinal tumors, inguinal lymph node metastasis accounts for 7.5%–17.9% of cases of anal canal carcinoma.^17,18)^ This occurrence is attributed to the lymphatic drainage from the anal side of the dentate line, which directs lymph flow to the inguinal lymph nodes.^19)^ In contrast, the lymphatic spread of colon cancer to the inguinal lymph nodes frequently follows a sequential pathway from the principal lymph nodes at the root of the superior or inferior mesenteric artery, via the para-aortic and external iliac lymph nodes. Therefore, a deviation from this conventional lymphatic pathway is required for solitary inguinal lymph node metastasis from colon cancer to occur.^9)^ Only 10 cases of inguinal lymph node metastasis originating from colon cancer have been reported to date. The proposed mechanisms of metastasis encompass the following: lymphatic spread via the lymphatic channels surrounding the inferior epigastric artery due to abdominal wall invasion in 5 cases,^2–4,7,8)^ direct extension from peritoneal dissemination in 1 case,^5)^ metastasis via intra-abdominal structures in 1 case,^10)^ iliac lymph node involvement in 1 case,^6)^ and an unknown mechanism in 2 cases.^1,9)^ In the present case, no evidence of peritoneal dissemination, abdominal wall invasion, or distant organ metastasis was observed, and the lesion was confined to a solitary inguinal lymph node. Therefore, previously reported mechanisms fail to fully explain the metastatic pathway in our patient. We further reviewed the clinicopathological characteristics of reported cases of inguinal lymph node metastasis from colon cancer, including the present case. With the exception of 1 case of signet-ring cell carcinoma, most reported tumors were well- to moderately differentiated adenocarcinomas, and no consistent tendencies were identified in regards to age, sex, primary tumor location, or the timing of inguinal lymph node enlargement (**Table 1**). Thus, based on available clinicopathological factors, it remains challenging to infer a definitive metastatic route for inguinal lymph node metastasis arising from colon cancer.

**Table 1:** Previously reported cases of inguinal lymph node metastasis from colon cancer

Author	Year of publication	Age	Gender	Location	Histological type	pStage	Time of detection of inguinal lymph node enlargement	Pattern of metastasis
Hakeem et al.^1)^	2009	60	W	Cecum	tub2	pT3N2M1	Same time	Unknown
Wu et al.^2)^	2010	37	M	Ascending	Tubular adenocarcinoma	No detail	Same time	Lymphatic spread along the inferior epigastric artery
Pisanu et al.^3)^	2011	62	M	Sigmoid	Adenocarcinoma	pT4N1M0	33 months after	Lymphatic spread along the inferior epigastric artery
Hara et al.^4)^	2013	67	M	Cecum	Mucinous carcinoma	pT4N1M0	3 years after	Lymphatic spread along the inferior epigastric artery
Kudou et al.^5)^	2015	85	M	Ascending	Moderately to well-differentiated adenocarcinoma	pT4N2M1	Same time	Direct extension from peritoneal dissemination
Kitano et al.^6)^	2017	83	W	Ascending	tub1	pT3N0M1	Same time	Iliac lymph node involvement
Tanabe et al.^7)^	2019	42	W	Sigmoid	tub1	pT3N2M0	2 years after	Lymphatic spread along the inferior epigastric artery
Alzahrani et al.^8)^	2020	59	M	Transverse	tub2	No detail	Same time	Lymphatic spread along the inferior epigastric artery
Neirouz et al.^9)^	2024	76	M	Ascending	Signet-ring cell carcinoma	pT4N2M0	7 years after	Unknown
Motamiez et al.^10)^	2024	35	W	Sigmoid	tub1	pT3N1M0	2 years after	Metastasis via intra-abdominal structures
Fujimoto et al. (current case)	2025	83	M	Transverse	tub2	pT4N0M0	1 year after	Unknown

M, men; tub1, well-differentiated adenocarcinoma; tub2, moderately differentiated adenocarcinoma; W, women

Mesh removal following inguinal hernia repair is frequently performed in cases of postoperative infection or chronic postoperative pain.^20)^ The recurrence rate of inguinal hernia following mesh removal due to mesh infection is 5%–14%, which is relatively low.^21–23)^ Hernia mesh implantation induces a local inflammatory response characterized by infiltration of inflammatory cells and fibroblasts, ultimately causing fibrous encapsulation.^20,24)^ This fibrotic remodeling reinforces the surrounding tissue, likely obviating the necessity for additional repair following mesh removal.^23,25)^

In our case, to achieve an adequate oncologic margin and reduce the risk of residual disease, en bloc resection of the mesh was needed. The surrounding tissue demonstrated dense fibrotic scarring, and the structural strength of the inguinal region was preserved even after mesh removal. At the 4-month follow-up, no inguinal bulging or imaging findings suggestive of hernia recurrence were observed; however, long-term follow-up is required. To the best of our knowledge, this is the first reported case of mesh removal necessitated by tumor involvement. Currently, no established treatment strategies exist for such cases. Similar to cases in which mesh removal due to infection does not require additional reinforcement for preventing hernia recurrence, hernia prevention measures may also be unnecessary following mesh removal due to tumor involvement.

Similar to the mesh, the spermatic cord was also strongly adherent to the tumor, necessitating en bloc resection. The spermatic cord contains the vas deferens and the following 3 major blood vessels supplying the testis: the testicular artery, deferential artery, and cremasteric artery. These vessels form anastomoses at multiple levels, contributing to collateral circulation that preserves testicular viability.^26,27)^ However, spermatic cord transection causes complete interruption of blood flow to the testis, potentially leading to testicular ischemia. Testicular ischemia may cause ischemic orchitis and subsequent testicular atrophy. To date, no reports have addressed the association between spermatic cord transection and testicular ischemia, and strategies regarding the necessity of prophylactic orchiectomy have not been established. Although some reports have described orchiectomy performed for preventing ischemic orchitis following traumatic injury to the spermatic cord, others have documented successful preservation of the testis, indicating a lack of standardized strategies.^28)^ In our case, the testis was atrophic due to age-related changes, and the risk of ischemic orchitis was considered low; therefore, prophylactic orchiectomy was not performed. At 4 months postoperatively, no signs of testicular necrosis were observed, suggesting that even when spermatic cord transection is performed, prophylactic orchiectomy may possibly be avoided in older adult patients. Conversely, in younger patients, the risk may be higher, and prophylactic orchiectomy may be necessary. Comprehensive preoperative explanation and appropriate planning are crucial in such cases.

## CONCLUSIONS

In the present case, an extremely rare pattern of metastasis was observed, involving an isolated inguinal lymph node metastasis from transverse colon cancer. The metastatic mechanism remains unclear, and further accumulation of similar cases is required. In cases of inguinal lymph node metastasis with anticipated invasion into the mesh used in inguinal hernia repair or the spermatic cord, careful preoperative planning, encompassing a well-planned treatment strategy and detailed informed consent, is crucial.

## References

[1] Hakeem A, Khan M, Dugar N, et al. Inguinal lymphadenopathy secondary to adenocarcinoma of caecum: a case report. Open Med (Wars) 2009; 4: 131–3.

[2] Wu YY, Xing CG, Jiang J, et al. Carcinoma of the right side colon accompanied by Sister Mary Joseph’s nodule and inguinal nodal metastases: a case report and literature review. Chin J Cancer 2010; 29: 239–41. 20109359 10.5732/cjc.009.10106

[3] Pisanu A, Deplano D, Reccia I, et al. Unusual metachronous isolated inguinal lymph node metastasis from adenocarcinoma of the sigmoid colon. World J Surg Oncol 2011; 9: 128. 21999113 10.1186/1477-7819-9-128 PMC3206842

[4] Hara M, Takahashi H, Sato M, et al. Curatively resected isolated inguinal lymph node metastasis from cecum cancer: report of a case. Surg Today 2013; 43: 88–90. 23111463 10.1007/s00595-012-0380-9

[5] Kudou M, Murayama Y, Konishi H, et al. Peritoneal colon cancer metastasis to bilateral inguinal hernia repair sites: report of a case. Surg Today 2015; 45: 1053–7. 25319214 10.1007/s00595-014-1037-7

[6] Kitano Y, Kuramoto M, Masuda T, et al. Ascending colon cancer with synchronous external iliac and inguinal lymph node metastases but without regional lymph node metastasis: a case report and brief literature review. Surg Case Rep 2017; 3: 32. 28220469 10.1186/s40792-017-0309-z PMC5318304

[7] Tanabe T, Shida D, Tsukamoto S, et al. Metachronous metastasis to inguinal lymph nodes from sigmoid colon adenocarcinoma with abdominal wall metastasis: a case report. BMC Cancer 2019; 19: 180. 30813921 10.1186/s12885-019-5386-x PMC6391797

[8] Alzahrani AM, Alshehri A. Synchronous colon adenocarcinoma with right inguinal lymph node metastasis: a case report and brief literature review. Cureus 2020; 12: e11501. 33214971 10.7759/cureus.11501 PMC7671169

[9] Neirouz K, Mehdi TM, Mohamed G, et al. Is there a route for metachronous inguinal lymph node in colonic cancer? A case report. J Surg Case Rep 2024; 2024: rjae024. 38389518 10.1093/jscr/rjae024 PMC10881287

[10] Motamiez A, Zedan A, Samir M, et al. Isolated inguinal lymph node metastasis from colon cancer: a case report. Indian J Surg Oncol 2024; 15: 525–7. 39239452 10.1007/s13193-024-01940-y PMC11371954

[11] Bergquist JR, Thiels CA, Spindler BA, et al. Benefit of postresection adjuvant chemotherapy for stage III colon cancer in octogenarians: analysis of the National Cancer Database. Dis Colon Rectum 2016; 59: 1142–9. 27824699 10.1097/DCR.0000000000000699

[12] Sadahiro S, Sakamoto K, Tsuchiya T, et al. Prospective observational study of the efficacy of oral uracil and tegafur plus leucovorin for stage II colon cancer with risk factors for recurrence using propensity score matching (JFMC46-1201). BMC Cancer 2022; 22: 170. 35168560 10.1186/s12885-022-09267-z PMC8845390

[13] Shibutani M, Maeda K, Kashiwagi S, et al. Effect of adjuvant chemotherapy on survival of elderly patients with stage III colorectal cancer. Anticancer Res 2021; 41: 3615–24. 34230158 10.21873/anticanres.15150

[14] U.S. Department of Health and Human Services. Common Terminology Criteria for Adverse Events (CTCAE) version 5.0. National Cancer Institute. 2017. https://ctep.cancer.gov/protocoldevelopment/electronic_applications/docs/CTCAE_v5_Quick_Reference_8.5x11.pdf. Accessed July 15, 2025.

[15] Guan X, Ma Z, Yang J. Occult ovarian high-grade serous carcinoma presenting as isolated inguinal lymph node metastasis: a case report and literature review. Transl Oncol 2025; 55: 102371. 40179456 10.1016/j.tranon.2025.102371 PMC11999682

[16] Zhang LL, Wang YS, Zheng A. Ovarian cancer presenting with isolated inguinal lymph node metastasis. Asian J Surg 2023; 46: 5147–8. 37451892 10.1016/j.asjsur.2023.06.112

[17] Yamada K, Saiki Y, Sugimoto K, et al. The inguinal lymph nodes as regional lymph nodes in anal canal adenocarcinomas: a nationwide database analysis in Japan. Surg Today 2024; 54: 1505–13. 38958723 10.1007/s00595-024-02888-w

[18] Torigoe T, Hirata K, Yamada K, et al. Metastatic status and dissection effect of regional/extraregional lymph nodes in Japanese patients with squamous cell carcinoma of the anal canal: a multicenter retrospective cohort study. J Anus Rectum Colon 2025; 9: 33–40. 39882228 10.23922/jarc.2024-039 PMC11772795

[19] Lengelé B, Scalliet P. Anatomical bases for the radiological delineation of lymph node areas. Part III: pelvis and lower limbs. Radiother Oncol 2009; 92: 22–33. 19095323 10.1016/j.radonc.2008.11.007

[20] Arnaud JP, Eloy R, Adloff M, et al. Critical evaluation of prosthetic materials in repair of abdominal wall hernias: new criteria of tolerance and resistance. Am J Surg 1977; 133: 338–45. 139832 10.1016/0002-9610(77)90542-6

[21] Rehman S, Khan S, Pervaiz A, et al. Recurrence of inguinal herniae following removal of infected prosthetic meshes: a review of the literature. Hernia 2012; 16: 123–6. 21858435 10.1007/s10029-011-0873-2

[22] Fawole AS, Chaparala RPC, Ambrose NS. Fate of the inguinal hernia following removal of infected prosthetic mesh. Hernia 2006; 10: 58–61. 16284700 10.1007/s10029-005-0031-9

[23] Akyol C, Kocaay F, Orozakunov E, et al. Outcome of the patients with chronic mesh infection following open inguinal hernia repair. J Korean Surg Soc 2013; 84: 287–91. 23646314 10.4174/jkss.2013.84.5.287 PMC3641368

[24] Bellón JM, Bujan J, Contreras L, et al. Macrophage response to experimental implantation of polypropylene prostheses. Eur Surg Res 1994; 26: 46–53. 8137846 10.1159/000129317

[25] Ito H, Matsumoto K, Terauchi T, et al. Delayed mesh infection after inguinal hernia repair: a case report. J Surg Case Rep 2021; 2021: rjab399. 34567517 10.1093/jscr/rjab399 PMC8458906

[26] Martinoli C, Pastorino C, Bertolotto M, et al. Ecografia del testicolo con color-Doppler. Tecnica di studio e anatomia vascolare., Color-Doppler echography of the testis. Study technique and vascular anatomy, in Italian with English abstract Radiol Med 1992; 84:785–91 (Radiol Med). 1494684

[27] Dellabianca C, Bonardi M, Alessi S. Testicular ischemia after inguinal hernia repair. J Ultrasound 2011; 14: 205–7. 23396995 10.1016/j.jus.2011.10.004 PMC3558245

[28] Takasu A, Morita K, Kaneko N, et al. Spermatic cord injury associated with blunt trauma. Am J Emerg Med 2005; 23: 806–7. 16182992 10.1016/j.ajem.2005.02.053

